# Negotiating Your Way Through the Vigilance Agreement Maze—is There a Better Way?

**DOI:** 10.1007/s43441-023-00513-5

**Published:** 2023-06-03

**Authors:** Catherine Corbel-Ecalard, Jean Kilgour-Christie, Weronika Trun, David J. Lewis

**Affiliations:** 1 Amgen Inc., Thousand Oaks, CA USA; 2 Global Drug Development, Novartis Pharma GmbH, Wehr, Germany; 3 Bayer AG, Medical Affairs & Pharmacovigilance, Muellerstr. 178, 13353 Berlin, Germany; 4 School of Life and Medical Sciences, University of Hertfordshire, Hatfield, Herts AL10 9AB UK

**Keywords:** Pharmacovigilance agreement (PVA), Safety data exchange agreement (SDEA), Contract negotiation, Process mapping, Pharmacovigilance partnerships, Glossary, PVA lifecycle

## Abstract

The pharmacovigilance agreements (PVA) landscape has evolved over recent decades with rapid growth in the number and complexity of partnerships, mergers, and acquisitions between pharmaceutical companies. Simultaneously there has been increasing scrutiny from regulatory authorities. Detailed regulations and guidance are lacking in this domain; hence companies have developed their own processes, templates, and tools, which have headed in different directions. Where feasible, marketing authorization holders (MAHs) have written contracts based on mutually understood requirements. Currently, MAHs are striving to find optimal solutions that safeguard patients, and in turn, support pharmacovigilance compliance. Through the TransCelerate BioPharma consortium MAHs are seeking simplification and efficiencies, to optimize the process of developing contractual agreements for pharmacovigilance. A survey of MAHs confirmed the perceptions above, and the need for efficient solutions to help navigate through the maze of complexity. The authors have led the development of tools and techniques to enable partnership between MAHs, and ultimately to support patient safety.

## Introduction

Pharmacovigilance agreements (PVA) and alliance partnership management is an area of constant challenge across Pharmacovigilance (PV) departments. The landscape is continuously evolving, with an ever-increasing number and complexity of partnerships throughout the industry. In the past, companies predominantly managed the development of medicinal products in-house. However, in today`s environment with increased numbers of mergers and acquisitions there are agreements which can consist of multiple parties and involve complex regulatory operating models. Collaboration is required at different stages of the product lifecycle from early development to post-marketing, and each phase poses different challenges. Industry has expanded over a larger network of vendors and wider range of medicinal products, devices (medical devices, software as a medical device), and combination therapies, but also advanced therapy medicinal products (ATMP), vaccines, radioligand, cell and gene therapies, etc. The need for rapid development of effective therapies for SARS-CoV-2 manifested in reduced development timelines along with the need for fast negotiations and approvals of PVAs across companies.

In addition, there is an increase in the scrutiny by regulatory authorities with the incremental expectation of both pharmacovigilance compliance and of regular, risk-based auditing and inspecting. There is not only an expectation but also a demand from all parties impacted by PV activities, and specifically regulatory authorities, to ensure compliance with all operational- and process-based regulations. There is a regulatory requirement for companies to establish a PVA if they are collaborating with other companies on post-marketed or clinical compounds [GVP VI.B.7/C.1.2/C.2.2]. Although regulations are available for specific PV activities (see specimen list which is not intended to be all-inclusive [1–10]), there is no overall regulation or guidance for a PVA template, nor are there other requirements that addresses this topic.

As companies set up partnerships, many aspects of the PVA must be negotiated and agreed upon to be able to operationalize and meet regulatory requirements for pharmacovigilance. Consequently, companies have developed their own internal processes, templates, and tools to manage PVAs. Such tools and processes can vary immensely across companies and may lead to misalignments and often convoluted or prolonged negotiations. Furthermore, there is a risk of a disconnect between information in the PVA and the operationalization of the agreement’s requirements. These challenges may delay the timely start of clinical trials and/or marketing authorizations of products and potentially influence patient safety and choice of treatment by healthcare providers.

There is a need for simplification and streamlining of structured processes and content that can be used by companies of differing sizes and varied portfolios when creating PVAs. The TransCelerate PV Agreements Optimization (PVAO) Initiative was established to develop tools to optimize PVA processes and create efficiency for all parties. This initiative focused on addressing four challenges for PVA development: (1) Process Map, (2) Table of Contents (ToC), (3) Glossary/definitions, and (4) Timelines for Safety Data Exchange. An integrated set of solutions was developed to address these challenges. This paper provides an overview of the first three solutions and their development.^1^

## Methods

This project involved large to medium-sized pharmaceutical companies from the TransCelerate BioPharma consortium that comprises 20 member companies. In order to gain a preliminary understanding of the PVA landscape, an initial list of five questions (Table 1) was prepared and distributed to the companies working on the PVAO Initiative. Anonymized and aggregated results were used to develop the integrated solutions.

**Table 1:** Discussion questions

1	Please provide a list of commonly used terms that are defined in your agreements. Please do not provide definitions
2	Please provide a list of commonly used table of contents headers that are used in your agreements
3	Please provide a list of commonly used standard timelines that are used in your agreements (e.g., 7-day report, 15-day report, Periodic Safety Update Reports (PSURs), Developmental Safety Update Reports (DSURs), Periodic Benefit Risk Evaluation Reports (PBRERs))
4	Please provide a list of terms for which differing interpretations have negatively impacted your agreement negotiations
5	Please provide a list of Table of Contents headers for which differing interpretations have negatively impacted your agreement negotiations

Each work stream within the PVAO Initiative (Process Map, ToC, Glossary) used different methods as outlined below.

### Process Map

The process map was drafted de novo by the PVAO initiative’s subject matter experts based on their knowledge and experience. The objective was to provide a visualization of the business process across the PVA lifecycle. Activities and steps were identified along with a sequence described in a flowchart. In order to clarify the process map, the flowchart was divided into four phases with associated steps. The key elements of each phase and step were described to outline the scope and importance of conducting key activities, including potential risks that can be mitigated.

The process map was then evaluated by the PVAO initiative team to identify inefficiencies throughout the PVA lifecycle. Identified inefficiencies triggered the creation of additional tools to support systematic PVA negotiations or facilitate a streamlined PVA review process. Finally, the process map was transferred into a web-based interactive platform with embedded customizable tools on the TransCelerate web page (Table 2).

**Table 2:** Process Map—potential areas for improvements in the process and embedded tools

Bottlenecks along the PVA lifecycle	Tools embedded in the Process Map to address the bottleneck
Deficiencies in communication and general understanding of the PVA-related processes	Downloadable “Process Map” graphic and the online interactive version with detailed description of the process
Late involvement of PVA responsible functions in new business partnerships	Pre-PVA Involvement of Pharmacovigilance (PV) Function tool
Inefficiencies in the negotiation process	Systematic Approach to PVA Negotiation tools (Kick-off Agenda, Gantt Chart, Five Steps for Efficient Negotiation Graphic)
Challenges in process and documentation of the periodic PVA review	Periodic PVA Review: Checklist & Documentation tool

### Table of Contents

Responses to the questions in Table 1 produced a list of headings that were reviewed for duplicate/similar terms, groups of terms, synonyms, and common heading groups. The terms were ordered by topic, and then grouped into four categories, in order to provide a modular approach to the PVA. The four modules are: (1) Background; (2) PV Activities; (3) Cooperation and Support; and (4) Appendices and Annexes. Within each category, key headings were identified (i.e., key topic headings) and, in some instances, sub-categories were also identified.

In addition, two filters were identified: (1) Region (European Union [EU]/European Economic Area [EEA], United States [US], and Japan), and (2) type of contract with respect to developmental stage (i.e., post-marketing, clinical trial). Furthermore, additional clinical trial sections and/or addenda were developed for specific situations such as clinical trial supply agreements, vaccines, and combination products. Medical device agreements were excluded.

For each heading in the ToC, the team identified a list of “points to consider” (PtC). Points to consider were proposed instead of template text to provide understanding, context, and flexibility in adoption. The PtC list is an extensive, but not exhaustive, list of items for the user to consider while developing/updating a PVA template or the PVA itself. These PtCs may be used according to the type of PVA, product, region, and other factors. The PtCs are based on regulatory references, information from inspection findings^2^ and the expertise of the subject matter experts over many decades and the experience of the management of a broad range of partnerships.

Several regulatory authority websites [Appendix 1] and the Medicines & Healthcare Products Regulatory Agency (MHRA) Good Pharmacovigilance Guide [11] were reviewed for inspection findings to inform the PtCs. See Appendix 1. The information obtained from the different inspectorates is provided in Appendix 2.

Once all modules were nearing completion, the TransCelerate team engaged with external stakeholders (defined as “stakeholder engagement”) to solicit feedback on the draft documents. Stakeholders were chosen to address all aspects from the ToC, i.e., to cover a broad range of industry associations from the US, EU, and Japan, companies of different sizes and covering post-marketing and clinical studies and a broad range of product types. The draft modules were made publicly available for about eight weeks on the TransCelerate website and external industry associations (EFPIA, JPMA, PhRMA, Medicines for Europe, TransCelerate’s Contract Research Organization (CRO) Forum) were contacted to solicit comments. Additionally, a focused campaign to obtain feedback and comments was initiated on LinkedIn to a targeted global audience of PV experts.

Comments received from member companies and external engagement were reviewed and addressed as appropriate.

### Glossary

**Figure 1:**
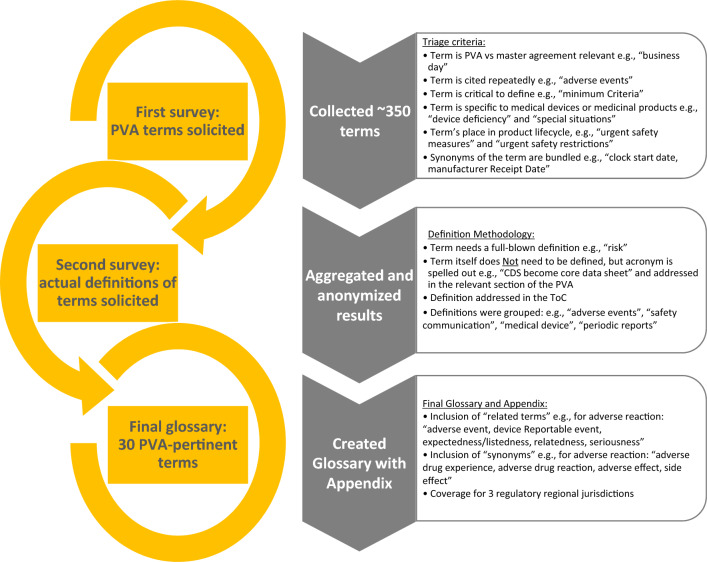
Development of the glossary.

As depicted in Fig. 1, data from the initial survey questionnaire produced a list of over 300 terms. These terms were triaged based on relevance to a PVA versus other ancillary agreements, the frequency of use of the term, the criticality to define the term, whether the term was pertinent to medical devices or medicinal products, and the term’s place in a product lifecycle (e.g., clinical, post-marketing). Synonymous terms were grouped together.

A second survey was used to solicit definitions for the terms. Results were aggregated and anonymized, and once the most common PVA terms were identified, draft definitions were developed using a collegial review and evaluation by the glossary team. The team determined if (1) a definition qualified for the incorporation into the glossary section in the PVA; (2) if a simple naming of the term (or acronym) was sufficient (e.g., core data sheet, periodic safety update report) in the body of the PVA; and/or (3) the definition would be addressed in the ToC. Definitions were then grouped by categories (e.g., adverse events [AEs], safety communication, medical device, and periodic reports).

The development of the glossary included the consideration of regulations in the US, EU, and Japan including ICH or, in the absence of regulatory language, definitions were developed based on a combination of text from individual companies, the team’s subject matter expertise, and on the team’s collaborative research. During this exercise, it was determined that other terms that were similar in scope and/or may be helpful to provide additional context for the user would be collectively referred to as “associated terms” in the glossary.

### Literature Search

A literature search was performed using Ovid Databases: MEDLINE(R) ALL 1946 to May 03, 2021 and Embase 1974 to May 03, 2021. The following key words were searched: Pharmacovigilance contract, Pharmacovigilance agreement, Vigilance agreement, Vigilance contract, Safety data exchange, Pharmacovigilance licensing, Pharmacovigilance co-development/co-marketing agreement, PVA lifecycle, PV alliance, PV obligations, Pharmacovigilance partnership, and Pharmacovigilance contract framework.

## Results

### Process Map

**Figure 2:**
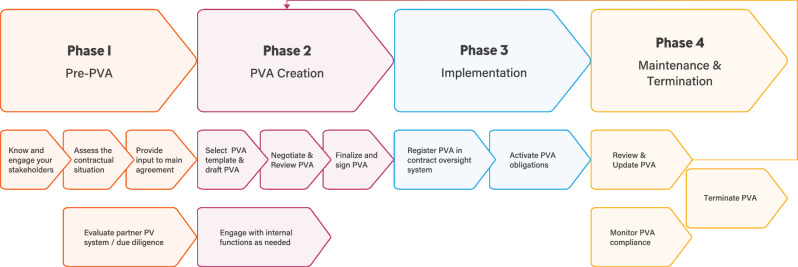
Process Map.

PVAs follow a lifecycle beginning with the establishment of a new partner collaboration and ending with the termination of PV responsibilities in that partnership. Based on such lifecycle a flexible and nimble framework “process map” (Fig. 2) was developed and published in the online version with embedded tools and helpful considerations [12].

The process map provides a phased approach to the overall PVA process and allows companies to follow a pathway through the maze and identify potential blind alleys, cul-de-sacs, or dead ends. The four sequential phases: (1) Pre-PVA, (2) PVA creation, (3) PVA implementation, and (4) PVA maintenance and termination were supported with helpful considerations that foster efficiency and compliance in PVA-related activities.

- **Phase 1 “Pre-PVA”** includes engaging with internal main contract owners, reviewing, and providing input to the main or overall partnership agreement, and performing due diligence or partner qualification. Having well-established pre-PVA procedures may support organizations in building a foundation for the PVA process by providing clarity for key stakeholders and the contractual strategy.
- **Phase 2 “PVA Creation”** includes drafting, negotiating, finalizing, and signing the PVA while engaging with internal functions. Development and negotiation of the PVA is key for all contract parties to ensure their PV system requirements and regulatory compliance obligations can be fulfilled. In this phase companies strive for efficiency, accuracy, and quality, since the PVA finalization may be directly linked to clinical trial start, product launch, or asset transfer activities.
- Successful **PVA implementation (Phase 3)** can be defined when stakeholders begin to comply with the terms of the signed agreement. Failure to implement a PVA correctly may result not only in compliance issues, but also delay the implementation of the license/Marketing Authorization/product launch or clinical trial.
- **Phase 4 “PVA Maintenance and Termination”** involves reviewing, updating, and continuously executing current PVA obligations. The PVA lifecycle may end with the termination of an agreement or could return to Phase 2 upon PVA updates and/or renegotiations. Correct implementation and maintenance of executed PVAs support more effective PVA oversight. In addition, the monitoring of PVA obligations may help both parties comply with regulatory requirements.

While developing the process map, the team highlighted potential areas for improvements (Table 2) and developed tools to help optimize PVA business operations and simplify the overall process. These tools provide detailed, tangible suggestions or considerations for improving efficiency. The tools are embedded in the online version of Fig. 2 [12]. All tools may be downloaded and customized by the user. One example is the “Systematic Approach to the PVA Negotiation” tool for structuring the PVA negotiation process by using project management elements. The tool offers contract timelines planning, agreement on PV collaboration principles, and appropriate internal company stakeholder engagement, which may facilitate faster alignment. The “Periodic PVA Review Checklist and Documentation” tool helps facilitate a streamlined PVA review process. The “Pre-PVA Involvement of Pharmacovigilance (PV) Function” tool describes how to build internal communication with functions that are entering into new partnerships.

### Table of Contents

**Figure 3:**
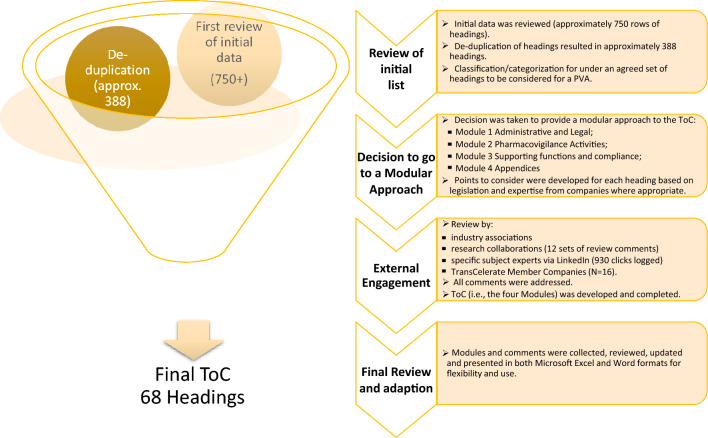
Overview of the summary of results for the preparation on the Table of Contents.

The diagram (Fig. 3) provides a summary of the results.

The final format of the ToC is modular consisting of four modules:

- **Module 1—Background**: This module addresses the terms of agreement, legal sections, and administrative information. Therefore, this module may also relate specifically to the individual company guidelines and legal requirements. Many of these issues may be appropriately addressed in the main agreement and the PVA, or solely the main agreement.
- **Module 2—Pharmacovigilance Activities**: Addresses the pharmacovigilance operational activities which are required for companies to include through regulation. It is not an exhaustive list, as required activities may depend on and be specific to the particular agreement and/or product.
- **Module 3—Cooperation and Support**: Addresses the quality management system, compliance, record management, governance, business continuity, audits, and inspections.
- **Module 4—Appendices and Annexes**: Identifies information which could be considered for appendices,

Each module comprises of a series of headings, some of which may have sub-headings. Each heading, or sub-heading, has a series of points to consider.

Due to the diversity of global regulations, the ToC does not include reference to PV regulations from all possible countries. Thus, the ICH regions (Europe, US, and Japan) were the points of focus. However, the document is written in such a way to make it useful in single countries, via addressing the applicable national regulatory requirements. With respect to the types of contracts, again allowing for flexibility, consideration was given to both pre-authorization (clinical trials) and the post-marketing setting. Depending on the type of agreement in such areas—in-licensing, out-licensing, distribution agreements, clinical trial supply agreements and others, the relevant headings and PtC can be used flexibly according to the needs of the parties. In addition, annexes outlining PtC for combination products and vaccines are included in the ToC.

In developing the ToC, the team adopted headings and a straightforward modular approach was developed to group similar topics and areas in the PVA together.

The intent was to provide a simple structure for companies, auditors, and inspectors.

**Figure 4:**
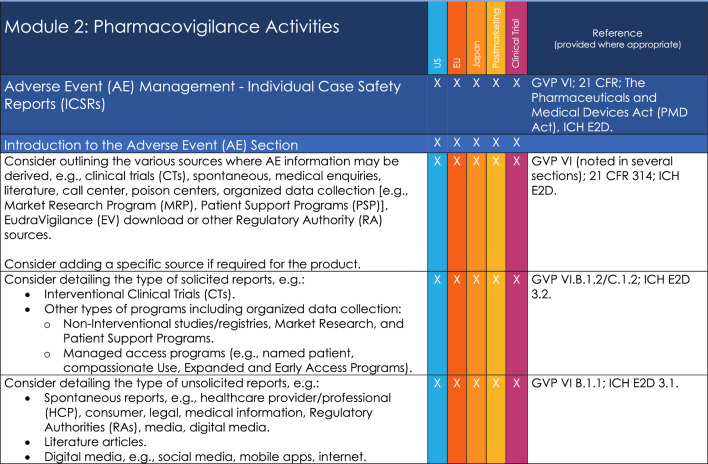
Example of one section of Module 2 Table of Contents.

An example of Points to Consider is provided on Fig. 4 and a list of the Key Headings for the ToC is provided in Appendix 3.

### Glossary

One company’s PVA terms often reflect the specific terms that are used throughout that particular company. Internal alignment for operationalization within one company may not necessarily align with the partner’s terms potentially resulting in circular discussions and extended negotiations. A large number of terms were returned in the responses to the survey of member companies. Several terms were specific to individual companies and many of the terms were considered by the team not to be essential for a PVA. In addition, the same words were being used for similar terms. A single harmonized glossary providing unified definitions enables companies to align more easily with the basic principles within the PVA, and may facilitate simultaneous implementation by the partner companies.

The glossary provides definitions for 30 key PVA terms. The glossary may, therefore, be particularly helpful when a PVA is required in a short timeframe as negotiating parties may elect to utilize the definitions so that more time can be allocated to negotiations on other important parts of the PVA, e.g., timelines. The member survey results also highlighted variations in how glossary terms are captured in PVAs: not all companies include a glossary/list of definitions, and the number of terms varies. The glossary is intended to be flexible for mature or novel PVA development processes and could potentially reduce PVA negotiation time.

In addition to the terms and definitions in the glossary, supporting documentation was developed as part of the solution and compiled into an addendum. The addendum provides comprehensive information about the references that were used to support the creation of the Glossary definitions. Furthermore, it provides cross-referencing of terms through hyperlinks that are integrated in the document, a listing of synonymous terms where applicable, and associated terms, which may provide additional context to the user.

### PVA Optimization Suite

**Figure 5:**
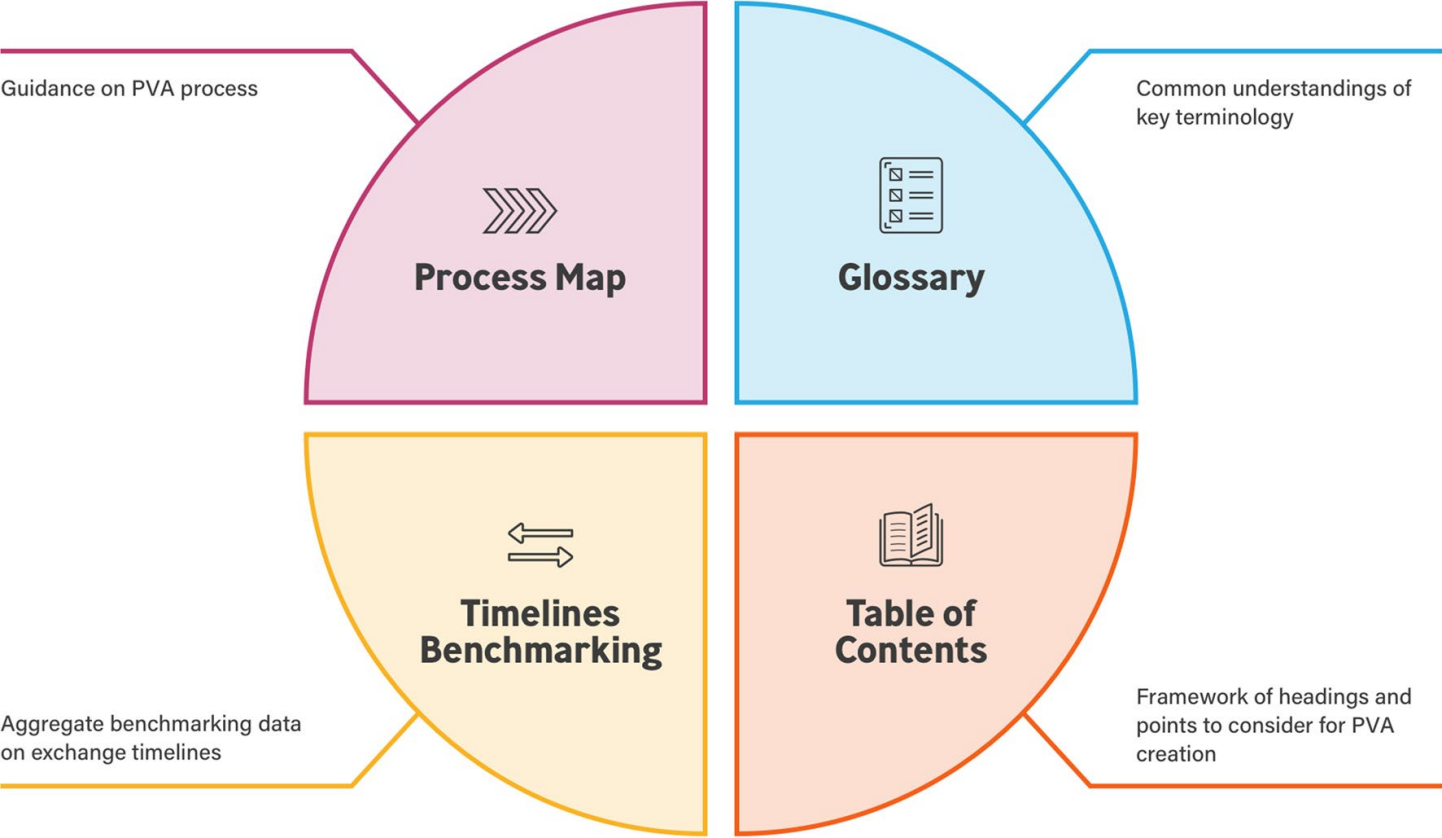
PVA optimization suite.

The Process Map, Table of Contents, and Glossary Solutions are made available in a web-based interactive toolbox [12]. A fourth solution (Timelines Benchmarking) remains under development at the time of this paper. All solutions were designed to complement each other, and several can be downloaded and customized according to the needs of each legal entity (Fig. 5)^3^.

## Discussion

### Root Causes of Complexity

Specific challenges were related to a variety of scenarios as noted in the introduction including an example of small biopharmaceutical companies working across territories. The challenges inherent here are not limited to the joint working of small and large companies, but also related to the limited experience of third countries with other territory regulation and requirements placed concerning PVAs.

Increased complexity of PVAs and limited information in both the public domain and regulations prompted a desire for more efficient approaches. The member companies moved through a PVA maze by collecting and analyzing the information from the work streams as described in the results section.

Several different questions arose, including:

“What is defined as a PV Agreement?”

“What types of contractual relationships require a PVA?”

“Are PVAs only required for the full sharing of commercial sales between two MAHs?”

“Does an agreement with a vendor providing outsourcing activities require a PVA?”

“Does an agreement with a contract research organization (CRO) for clinical trial supplies require a PVA?”

“Should clauses covering pharmacovigilance responsibilities be located in the main agreement or in an annex specific for pharmacovigilance?”

And finally, “Should we refer to a PVA or safety data exchange agreement (SDEA)—are they the same?”.

Our research showed that most problems were encountered when negotiating the details of PVAs. Even after PVA execution, further difficulties are noted during the operationalization of the contractual commitments due in some measure to the variety and complexity of agreements required multiple customizations of existing in-house processes.

### Rationale for the Toolbox

Literature research was conducted which confirmed that there was little information regarding the PVA process in the public domain. Most of the relevant publications were dated before 2013, with the majority published between 2000 and 2010 [13–18]. Although some information remains applicable to today`s PV systems, the complexity described above was not present, hence it was not considered.

Sixteen TransCelerate Biopharma member companies (with additional input of stakeholder engagement) attempted to find potential efficiencies. The current complex PVA landscape is managed across differing company frameworks that often allow agreement on the general principles but may sometimes lead to disagreement and contentious discussions concerning the details. The initiative supported a healthy and constructive discussion on the most ambiguous areas in the PVA negotiation and challenging types of PVAs. As a result, solutions offering a flexible and agile framework were proposed in the form of a PVAO Suite containing a process map, table of contents, glossary and timelines benchmarking to support the facilitation of earlier issue resolution and more efficient collaboration between the parties negotiating the PVA.

### PVA Optimization Suite

The management of PVAs requires time, resources, and expertise to maintain regulatory compliance. A goal was to illustrate the PVA lifecycle and provide considerations and tools to support organizations that engage in PVA activities to simplify, optimize, and improve efficiencies in the PVA process.

The PVA journey travels through a maze of different parties, including internal and external stakeholders, various geographic regions, associated regulations, and product variations. All of the above are further complicated by the existence of fundamental operational differences between pharmaceutical companies. Negotiations often involve circular discussions with the revisiting of different elements within the agreement process and iteration until the goal of a signed contract is achieved. By adoption of the process map, organizations may be better able to navigate their way through the required steps and reach the desired goal without experiencing repeated delays.

Embedded tools within the Process Map intend to address potential areas for improvements (Table 2) and to help optimize PVA management. Companies may have multiple functions that are entering into new partnerships or due diligence matters and building sustainable internal communication between PV and these functions may become key to success for early involvement of PV. The tool “Pre-PVA Involvement of Pharmacovigilance (PV) Function” offers support to organizations in fostering internal communication. Bottlenecks along the PVA lifecycle can be managed by implementing structured processes. The “Periodic PVA Review Checklist and Documentation” tool suggests how to streamline PVA review process, whereas “Systematic Approach to the PVA Negotiation” tool proposes structuring the PVA negotiation process by using project management elements. These tools provide tangible process descriptions that may be valuable guidance for improving efficiency and support compliance.

At times, during negotiations or even during the operationalization of the PVA, partners may experience misalignment and potentially compliance issues over the meaning of a term and its implications for the success of the implementation of the PVA. Defining PVA terms that are meaningful and unambiguous solidifies the respective parties’ comprehension rather than allowing them to meander toward dead ends. The value of offering commonly understood definitions for terms such as “awareness date,” “minimum criteria,” “day zero,” “invalid,” or “incomplete case” can result in greater agreement clarity, clearer expectations, and may help reduce short- and long-term operational hurdles.

A goal of this project was to develop a comprehensive and flexible framework for managing PVAs that may help to reduce the time taken for PVA development and support compliance with regulations and to the PVA. Two partner companies using information from the ToC or starting negotiations with the same understanding of the PVA definitions can potentially ultimately reduce the negotiation time, which could allow the focus to be spent on specific topics of relevance. Moreover, a transparent and documented end-to-end process has the possibility to avoid PVA process issues and faster resolution thereof.

These tools aim to enhance efficiency in PVA development and simplify processes which could then ultimately support the training of personnel and conduct of audits/inspections. The structure and processes offered by these tools may provide a more time-efficient means of ensuring that appropriate auditing is feasible for all partners, and could ultimately reduce the number of findings and observations.

### Next Steps

All stakeholders are striving for regulatory compliance, ideally accompanied by efficiency gains, operational excellence, and achieved in a collaborative manner. In the future, such tools may help contractual partners to achieve these goals.

The PVAO suite was designed to support greater internal streamlining of the process, enabling and providing further opportunities. The authors noted that there are other issues in the PVA area for further investigation, which are outside the remit of the current project. It is considered that PVAO solutions provide a foundational platform that could further expand into areas such as, but not limited to, automation of the PVA development process and model contracting.

An important area of the PVA lifecycle is the monitoring of PVA obligations during the maintenance phase of the PVA lifecycle, as this allows early identification of potential non-compliance. A strong monitoring program can inform the risk-based audit strategy. Companies struggle with defining the level of granularity when setting up the monitoring processes, but also with the lack of guidance or the availability of methods and tools.

Audits and inspections may benefit from the use of the PVAO suite in terms of transparency of the process with a recognizable structure and framework for the PVAs. Audits and inspections are a challenge in terms of time, resources, and findings and the use of such tools may help to reduce such challenges.

There is a global increase in the number of medical device regulations [19–29]. As a result, the development of device vigilance agreements is increasingly challenging for many companies. The need for more guidance and solutions is manifest. Overall, the use of such tools may potentially reduce the time spent on drafting and negotiating PVAs and enable a concentrated focus on specific topics of importance.

## Conclusion

In order to navigate effectively through the vigilance agreements maze, there is a need for simplification and process streamlining to facilitate regulatory compliance, and meet auditors’ and inspectors’ expectations. The TransCelerate PVAO Initiative was established to support this effort in relation to PVAs. The pharmaceutical industry has expanded to encompass a network of vendors, partners, and a wider range of medicinal products, devices, and combination therapies.

TransCelerate’s PVAO suite of solutions aims to support more efficient and effective collaboration between partners and provides a step forward in rationalizing a complex area. Faster and more efficient management of PVAs can potentially help to mitigate delays to patient access because quicker PVA execution may support earlier clinical trial start, managed access programs, product launch, or asset transfer activities. This suite of tools is not a one-size-fits-all solution; rather, it lays a foundation that can be further elaborated upon and expanded to cover other elements of the PVA business, such as devices and model contracting, and obligation monitoring. Further research, outside the remit of the current project, could be to embrace technology and digitalization advancements. It is clear that if automated processes are an option for the creation and management of PVAs, there is a genuine possibility that artificial intelligence (AI) can aid governance of PV systems.

Our aim is to suggest new, efficient ways of working, to simplify navigation through to completion of new PVA partnerships. We recognize that there is a need to provide agile solutions based on a nimble framework and accompanied by flexible tools. Fostering better and more effective collaboration between companies can result in improved compliance and efficiency that helps to support patient safety and potentially facilitates earlier access to medicines.

## Appendix 1

Health authority inspectorates reviewed for potential PVA observations

**Table:**

Health authority	Website	Time period	Review comment
MHRA	*Pharmacovigilance inspection metrics, 2009 to present—GOV.UK (*www.gov.uk*)*	2013 to 2021	“PV Contract” findings are categorized in the QMS Section. “Safety Data Exchange Agreements” findings are categorized in the Collection and Collation of Adverse Drug Reactions Section
FDA	*Inspection Observations | FDA*	2014 to 2020	The Inspection Observation Summaries were reviewed
TGA	*Pharmacovigilance Inspection Program metrics report: Jan–Dec 2020 | Therapeutic Goods Administration (TGA)*	Sep 2017 to Dec 2020	Observations for PVAs seen in Sections: Collection and collation of adverse drug reactions Safety agreements and contracts between vendors and partners
EMA	*Pharmacovigilance Inspectors Working Group | European Medicines Agency (europa.eu)*	2014 to 2020	Information from supervisory inspections in EU/EEA is summarized in the Inspectors Working Group Annual Report. This provides a high-level summary and lists any topics for discussions in the section: In relation to medicinal products for human use

*EEA *European Economic Area, *EMA *European Medicines Agency, *EU * European Union, *FDA *Food and Drug Administration, *MHRA *Medicines & Healthcare products Regulatory Agency, *PV *pharmacovigilance, *PVA *Pharmacovigilance Agreement, *QMS *Quality Management System, *TGA *Therapeutic Goods Administration

.

## Appendix 2: Inspection Findings

Summary of Inspection findings for the Inspectorates Reviewed

**Table:**

Regulatory authority name	Results
MHRA	2020 to 2021—No PVA observations 2019 to 2020—2009 major findings were reported for deficient PSMF management, six of these findings were due to incomplete or incorrect information presented in the annexes to the PSMF, most notably for annexes B (lists of contracts and agreements) and H (list of products covered by the pharmacovigilance system). Exact numbers for PVA were not mentioned 2018 to 2019—No findings for PV Contracts. Nothing noted for SDEAs 2017 to 2018—Procedures, record management, training, PV contracts—noted one major and 11 minor findings and no further details were available 2016 to 2017—Critical findings: “There was no MAH oversight of pharmacovigilance activities still being undertaken by a service provider with whom contractual arrangements had been terminated.” “There were issues with oversight of PSP vendors, including deficiencies with the pharmacovigilance audit programme and contractual agreements.” “There were no measures in place to ensure appropriate set-up of MRPs outside of specific regions, to ensure that suspected adverse reactions are collected from these types of programmes. This included deficiencies with procedural documentation, PSMF content and contractual agreements with third-parties.” Minor findings were reported across 15 topic areas. The largest proportion of minor findings was in relation to the PSMF, followed by issues with pharmacovigilance contracts and agreements (12 observations) From this point backwards the MHRA data was presented in a different format and difficult to see if contracts and agreements were impacted 2015 to 2016 No findings in relation to contracts or agreements 2014 to 2015 No findings in relation to contracts or agreements 2013 to 2014 8% PV contracts—no further information available
TGA	Jan to dec 2020: Noted below are key parts of the section for context: *“Collection and collation of adverse drug reactions* A total of five major deficiencies and two minor deficiencies were identified for this topic which represented 19% of major deficiencies and 13% of minor deficiencies Deficiencies were identified in the following processes: Inclusion of relevant safety reporting provisions in all third-party contractual agreements prior to the service commencing Reconciliation of safety information with all possible sources Identification and collection of special situation reports Sponsors should maintain a pharmacovigilance system that allows them to identify, collect, and collate all information related to safety of their medicine from all possible sources. Sponsors should also exercise due diligence and develop procedures to collate accurate and complete reports of adverse drug reactions. It is important that sponsors maintain up-to-date contact information in all publically available sources and that processes are in place to monitor and act on information collected.” *Management of significant safety issues* “Sponsors are reminded that third party contractual agreements should include relevant safety reporting provisions that outline responsibilities related to the identification, management, and reporting of significant safety issues. There should be a process to ensure the maintenance of these provisions including for example, sponsor contact details.” Jan to Dec 2019: Under the section of “Reporting Serious ADRs” it was noted that: “Sponsors should periodically audit their pharmacovigilance agreements to ensure that business partners or contracted personnel are complying with their pharmacovigilance reporting obligations.” Sep 2017 to Dec 2018: Under Collection and collation of adverse drug reactions—to include deficiencies in the: safety agreements and contracts between vendors and partners
FDA	No findings from 2014 to 2020 directly related to PV contracts or agreements noted in the Inspection summaries
EMA (Supervisory Inspections)	IWG Annual Report: 2019 and 2020 Published 26 Nov 2021—Section: 4.6. In relation to medicinal products for human use—No discussions related to PV contracts/agreements 2018 Published 03 Jul 2020—12 major findings for “Contracts and Agreements” category for supervisory inspections 2017 Published 29 April 2020—No discussion topics related to PV contracts or agreements. However, the following discussion point was noted: issues arising in the case of marketing authorization transfer/transfer of ownership 2016 Published 08 Aug 2017—PV contract and agreements not listed in discussion topics 2015 Published 29 Jul 2016 No discussion topics related to PV contracts or agreements 2014 Published 12 Aug 2015—Discussion topics: approaches to pharmacovigilance inspections of different types of sites (supervisory authority headquarters), national affiliates, third parties/contractors) expectations on contractual arrangements and communication between MAH headquarters, affiliates and distributors; 2013 Published 06 Aug 2014—working on GVP implementation

The following inspection observations are highlighted in the MHRA 2009 Good Pharmacovigilance Practice Guide, Chapter 11 Contracts and Agreements (2):

- Signed contracts are not in place with a third party (e.g., starting a service prior to signing agreement).
- Not all services provided to marketing authorization holder (MAH) are in the contract.
- Timeframes to exchange PV information are not presented in the agreement.
- Company X has a 90-day timeframe for collection of non-serious AEs and unlisted AEs may be missing in the Periodic Safety Update Report (PSUR) if received one month prior to data lock (DL). Contract manufacturer agreements do not clearly identify manufacturers’ responsibilities related to safety.

## Appendix 3: Table of Contents Level 1 Headings

**Module 1: Background**

Background and Purpose

Main Agreement Term

PVA Termination

PVA Review

Severability/Precedence

Applicable Law/Legislation

General Principles

Confidentiality

Common Language

Mode of Communication Between Parties

Public Communication

Data Protection and Privacy

Assignment (Right to assign responsibilities without partner consent)

Liability

No waiver of rights

Conflict Resolution

Breach

Anti-corruption

General Legal Terms

**Module 2: Pharmacovigilance Activities**

QPPV

Pharmacovigilance System

Adverse Event Management—Individual Case Safety Reports (ICSRs)

Assessment of Seriousness, Causality/Relatedness, and Expectedness

Organized Data Collection

Website, Internet, Digital Media, and Other Similar Sources

Exchange of Individual Case Safety Reports (ICSRs)—Electronic Data Exchange

Safety Data Exchange Process Flow—Exchange Information

EV Download/ICSR Download from other Regulatory Authorities (RA)

Reconciliation

Literature

Signal Management

Aggregate Reports

Risk Management

Post-Authorization Safety Study (PASS) and Post-Authorization Efficacy Studies (PAES)

Benefit/Risk

Reporting to Regulatory Authorities (RA)

Regulatory Authority (RA) Queries

Reference Safety Information (RSI)

Clinical Trials/Studies

**Module 3: Support Activities or Cooperation & Support**

General Quality Management System Requirements

Pharmacovigilance System Master File (PSMF)

Amendments

Governance

Compliance/Compliance Oversight/Shortcoming, Deviation, and Corrective and Preventative Action (CAPA) Management

Regulatory Inspections

Audits

Training on SOPs and PV Requirements, Role Responsibilities/Requirements

Product Complaint/Quality

Record and Data Keeping

Cooperation and Support

Term/Termination

Delegation/Sub-contracting

Data Protection and Privacy

Business Continuity Planning

Confidentiality

**Module 4: Appendices and Annexes**

Abbreviations & Acronyms

Definitions

Product/Territory List

Responsibility Table

Contacts List

Routes and Methods of Communication

General Exchange Information (e.g., Timelines table)

Individual Case Safety Report (ICSR) Timelines

Study Information

Revision History (usually includes effective date, Version)

General Forms Section

General Legal Terms

PVA Signature

**Addendums:**

ADDENDUM 1: Listing of Headings (hyperlinks to document solutions)

ADDENDUM 2: Common Acronyms Used in the TransCelerate PVA Table of Contents

ADDENDUM 3: Termination Agreement Points to Consider

ADDENDUM 4: Specific Points to Consider for Products with Device Component

ADDENDUM 5: Specific Points to Consider for Vaccines

## Footnotes

1 Timelines for Safety Data Exchange is currently under development at the time of writing.

2 Inspection findings were not only based on anecdotal and experiential evidence, but from Regulatory Authority websites including MHRA, TGA, BfArM, and FDA.

3 Note: Timelines benchmarking is currently under development at time of writing.
